# A Decision Method for Construction Safety Risk Management Based on Ontology and Improved CBR: Example of a Subway Project

**DOI:** 10.3390/ijerph17113928

**Published:** 2020-06-01

**Authors:** Xiaoyan Jiang, Sai Wang, Jie Wang, Sainan Lyu, Martin Skitmore

**Affiliations:** 1 School of Civil Engineering, Hefei University of Technology, Hefei 230009, China; 2017170402@mail.hfut.edu.cn (S.W.); 2018170504@mail.hfut.edu.cn (J.W.); 2 School of Property, Construction and Project Management, RMIT University, Melbourne City Campus, Melbourne, VIC 3000, Australia; sainan.lyu@rmit.edu.au; 3 School of Civil Engineering and Built Environment, Queensland University of Technology, Brisbane, QLD 4001, Australia; rm.skitmore@qut.edu.au

**Keywords:** safety risk, ontology, CBR, similarity algorithm, correlation algorithm, subway

## Abstract

Early decision-making and the prevention of construction safety risks are very important for the safety, quality, and cost of construction projects. In the field of construction safety risk management, in the face of a loose, chaotic, and huge information environments, how to design an efficient construction safety risk management decision support method has long been the focus of academic research. An effective approach to safety management is to structuralize safety risk knowledge, then identify and reuse it, and establish a scientific and systematic construction safety risk management decision system. Based on ontology and improved case-based reasoning (CBR) methods, this paper proposes a decision-making approach for construction safety risk management in which the reasoning process is improved by integrating a similarity algorithm and correlation algorithm. Compared to the traditional CBR approach in which only the similarity of information is considered, this method can avoid missing important correlated information by making inferences from multiple sources of information. Finally, the method is applied to the safety risks of subway construction for verification to show that the method is effective and easy to implement.

## 1. Introduction

The construction industry is one of the most accident-prone sectors in the world [1], with an occupational mortality rate as high as 30–40% in many countries, making it the most deadly of all sectors [2]. Although many countries have made great improvements in safety, this industry still faces serious safety problems [3] due to the dynamic complexity of construction projects [4], a lack of experienced workers, and an uncertain weather environment [5]. How to effectively implement safety management has therefore become a common concern [3].

To improve safety management, risk identification and assessment are important steps [6]. However, most knowledge concerning safety risks is in various unstructured forms (e.g., expert experience, construction drawings, and construction organization design) [7,8], and the identification and evaluation of safety risks depends on the practical experience of domain experts [9]. In addition, due to the frequent mobilization of engineers and experts, and the inconsistency of communication between organizations and stakeholders, knowledge related to safety risks cannot be fully utilized, which sometimes impedes the implementation of safety risk management [10,11]. More importantly, construction safety accidents are composed of various elements such as time, space, people, events, features, and object status, which are often caused by a combination of multiple highly random, sporadic, and time–space complex risk factors. Therefore, an effective approach to safety management is to structuralize safety risk knowledge, then to identify and reuse it, and establish a scientific and systematic construction safety risk management decision system.

Case-Based Reasoning (CBR) technology has been increasingly applied to the field of construction safety management for retrieving, reusing, revising, and retaining previous research, and for providing the right solutions for a given problem [12,13,14,15,16]. Ontology, as a form of knowledge and information organization, combines certain domain knowledge and expression capabilities to support risk identification by structuring and standardizing safety risk knowledge [17,18], and plays an important role in the semantic representation and reuse of safety management knowledge. However, there are still some gaps in knowledge concerning the combination of ontology technology and CBR for construction safety risk management. In addition, recent studies have also demonstrated that attribute-based and distance-based similarity algorithms have their limitations in the CBR process [19].

In response, this paper proposes an improved method by integrating ontology and improved CBR for construction safety risk management. The main contributions of this study are as follows: firstly, by developing the construction safety risk domain ontology and combining ontology with CBR, a research framework for construction safety risk management decision-making is constructed, and the reliability and feasibility of the method is verified by a subway case study. Second, considering the similarity and correlation of cases, the CBR algorithm is improved to find the most similar cases. A hybrid similarity algorithm is used to improve the conventional similarity algorithm in considering the correlation relationship and proposing a comprehensive algorithm combining similarity and correlation, which greatly improves the accuracy and reliability of case reasoning. Finally, 83 cases of subway construction accidents in China from 2001 to 2019 are used to provide a model for similar studies in other countries and regions.

## 2. Literature Review

### 2.1. Construction Safety Risk Management

In the construction engineering field, risk management can be defined as a systematic process of identifying, analyzing, and responding to risks [20]. Table 1 lists a variety of different risk management methods used to estimate risks in construction engineering.

Furthermore, the complexity and uncertainty of the construction industry requires safety managers to use the latest technologies to ensure they cover as many foreseeable and unforeseeable safety risks as possible [21]. Therefore, smart safety management technologies have been developed in recent years. Ding et al. [22] proposed a metro engineering safety risk identification system (SRIS) based on construction drawings and applied it to graphic identification technology and risk identification automation technology to carry out risk assessment before construction. Zhang et al. [23] established a real-time model to identify possible safety risks among many potential risk factors. In addition, many studies use building information modeling (BIM) to identify safety risks. Kiviniemi et al. [24] identified potential safety hazards through BIM and determined the conditions and factors involved in safety risks. Kim et al. [25] proposed a risk source identification method based on BIM and a real-time location system of laborers, and ranked the safety risk factors and the proximity degree at a certain moment with real-time construction data.

Most importantly, previous studies involving construction safety risk management have mainly focused on risk identification and risk analysis rather than decision-making. Moreover, smart technologies in construction safety risk management are also in need of further examination.

**Table 1:** Construction safety risk management methods.

Construction Safety Risk Management		Method	Advantages	Disadvantages	Reference
Traditional methods	Safety risk factor identification	Expert interviews and questionnaires	Domain experts have rich professional knowledge, and the operation is simple, which is conducive to the rapid and accurate identification of risk knowledge	Subjective influence of interviewees	[26]
		Case analysis	Relevant to the actual situation, high credibility	Consider the influence of many different factors on the results of risk identification	[27]
		Expert interviews and interpretive Structural Models (ISM)	Turn expert expertise into intuitive, well-structured models	Strong subjectivity, the relationship between the elements in the system depends on people’s experience	[28]
	Safety risk analysis	Fault tree analysis (FTA) and event tree analysis (ETA)	The analysis is intuitive, clear and logical	Analysis of specific events with limitations	[29]
		Bayesian network and fuzzy fault tree analysis (FFTA)	Overcome limitations on the current probability estimation	The collection of safety-related knowledge relies heavily on domain experts	[30]
		Case analysis	Relevant to the actual situation, high credibility	Consider the influence of many different factors on the results of risk identification	[21]
	Safety risk response	The zonal-based approach	A tool for selecting risk response strategies	Only applicable when considering two risk criteria	[31]
		The trade-off approach	The desirable strategies can be selected among the candidate ones according to efficient frontier rule	Either consider only two factors or make trade-offs based on qualitative analysis	[32]
		The WBS (work breakdown structure)-based approach	When the analyzed activity is the actual one, risks are identified and strategies can be formulated directly associated with that activity or can be selected among candidate ones by an index of scope expected deviation	It is unknown whether the strategies obtained are an optimal solution to the strategy selection problem	[33]
		The optimization-model approach	Establish an optimization model to solve the risk response strategy selection problem	Can only be applied to small-scale projects	[34]
**Smart methods**		CBR	The most similar historical cases can be retrieved using the method	Requires that the information in the case base is very comprehensive	[35]
		Apply graphic recognition and risk Identification automation technology	Safety risks can be automatically identified from the knowledge database	High requirements for design codes and construction engineering experience	[22]
		Identify potential risk models in real time	Identify the possible safety risks factors in real time	The method of weight determination is more troublesome	[23]
		Building Information Modeling (BIM)	Facilitate the exchange and interoperability of project information management	System development requires a large number of domain experts to participate	[24]
		BIM and real-time location system of laborers	Identify hazardous areas in construction sites automatically	The accuracy of real-time location-tracking system positioning needs to be improved	[25]

### 2.2. Ontology Technology in Construction Safety Management

Ontology was originally a concept in the field of philosophy, and has been gradually applied to artificial intelligence (AI), knowledge management, libraries, and information. Though there are many different interpretations, most studies have the same view on the essence of ontology, the current authoritative definition proposed by Gruber [36] being that “Ontology is the conceptualization of terms and their relations in a domain”. Domain ontologies provide a set of terms for describing some domain, such as medicine, air campaign planning, or computer maintenance; they can be very large and include thousands of concepts [37]. At present, ontology technology is regarded as a method of organizing and representing knowledge concepts. Ontology formalizes knowledge through the classification of objects, attributes, and logical relationships between objects in a particular domain to facilitate information integration, retrieval, and reuse [38].

There are many methods for developing ontology models, including the Skeleton [39], KACTUS [40], TOVE [41], METHONTOLOGY [42], and Seven-step methods. The Seven-step method developed by Stanford University is used to build domain ontology, and is the most widely used ontology development method in studies throughout the world [8,43,44]. In comparison with other ontology development methods, the Seven-step method is considered to be a relatively mature and sound technology, as it focuses on the ontology development process and is highly applicable to construction work, as well as being especially suited to domain ontology development. It is therefore used in the present study as the development method for the safety risk ontology model.

With the infiltration of information technology and digital technology in multi-disciplinary fields, research into ontology technology has begun to appear in the construction industry [6,8,45,46]. In the field of construction safety management, ontology has also been used for the representation, sharing, exchange, and reuse of safety knowledge [6]. The interaction between safety ontology and BIM is also examined, and a prototype application of ontology-based job hazard analysis and visualization is made to further illustrate the applicability and effectiveness of the developed safety ontology [47]. Another study proposes an ontology-based semantic modeling method for construction safety knowledge that is combined with BIM for automated risk analysis [35]. Le et al. [48] combine ontology with social networks to propose a social network system for sharing construction safety and health knowledge to enhance communication between building project stakeholders and construction safety knowledge. Furthermore, other studies use ontologies to model and reuse construction knowledge for safety inspections [6], work hazard analysis [45], the emergency plan management of metro operations [49], and safety risk analysis [50].

### 2.3. Case-Based Reasoning (CBR) in Construction

Case-Based Reasoning (CBR) has enjoyed tremendous success as a technique for solving problems related to knowledge reuse [51]. One of the key factors in ensuring this success is CBR’s ability to allow users to easily define their experiences incrementally and to utilize their defined case knowledge when a relatively small core of cases is available in a case base [51]. Considering the construction industry’s large amount of historical experiences, it is unsurprising that the applicability and effectiveness of Case-Based Reasoning (CBR) has been demonstrated in various construction management areas, including construction tendering, bidding and procurement [52,53], construction contract management [15,54], international market selection [55], construction infrastructure maintenance [56], value engineering [57], onsite supervisory manpower [58], and construction cost estimation [59]. As a decision-support tool in construction, CBR is well suited to construction safety management (Table 2).

Typically, a case comprises the problem that describes the state of the world when the case occurred, and the solution comprises the derived solution to that problem and/or the outcome that describes the state of the world after the case occurred. There is no consensus in the CBR community over what information should be in a case. However, two pragmatic measures can be taken into account in deciding this: the functionality and the ease of acquisition of the information represented in the case [65]. Hence, CBR does not require explicit domain models, although attribute classifications and similarity calculations are important factors. Semantic similarity refers to the degree of similarity between two concepts and generally is 0 or 1. If the two concepts are interchangeable in any context, their similarity is 1; otherwise, if the two concepts cannot be replaced in any context, their similarity is 0.

It is generally agreed that the concept of a semantic similarity algorithm based on ontology can be divided into four categories: (1) semantic similarity based on attributes, (2) semantic similarity based on content, (3) semantic similarity based on distance, and (4) hybrid methods [66,67,68]. Some studies also suggest that semantic similarity should consider the hierarchy structure [66,67,69]. However, a case similarity algorithm based on attributes inevitably results in computational errors, for example, the stored case and the target case will commonly contain some missing or null attribute values in reality [70]. A content-based similarity algorithm determines the similarity of two classes by comparing the content information contained in the common parent node of the classes and ignores the content information contained in the keyword itself. The basic idea of a distance-based semantic similarity algorithm is to quantify the semantic distance between two conceptual words in the ontology tree classification system [71]. However, the main drawback of this method is to assume that the distance of all edges in the system is equally important in the ontology classification. Obviously, this assumption cannot be true—the importance of the edge being related to its location information and the type and strength of its association.

In most CBR applications, similarity is assessed based on attribute-value. For instance, cases with an internal structure require a similarity mechanism that considers structure descriptions of cases using similarity metrics that use these attribute values. As mentioned above, in the face of diverse attributes, practical experience in the development of CBR systems indicate that a simple attribute vector does not adequately represent the complexity of cases encountered in practice [72]. Therefore, the present study uses a hybrid similarity mechanism to determine the similarity of cases, which is applicable to the ontology currently constructed. The advantage of this algorithm is that it considers both the position information (distance, hierarchy) of the class in the ontology tree and the content information contained in the keyword itself.

Resnik [73] suggested that the assessment of similarity in semantic networks can be thought of as involving solely taxonomic links to the exclusion of other link types, although this admittedly excludes some potentially useful information. Similarly, in the CBR matching algorithm, compared with semantic similarity, semantic correlation is neglected by many studies, leading to the omission of much useful information. Semantic similarity and the semantic correlation between two objects has long been a basic problem in the field of data mining and knowledge management, but they are two different concepts [74]. Semantic correlation refers to the degree of the interrelationship between two concepts. It is very common to have no similar relationship between two concepts of semantic correlation but a correlation relationship may be formed by some other reasons. Semantic similarity is the aggregation of concepts, while semantic correlation is the combination of concepts. Resnik [73] explained the difference between similarity and correlation using the example of cars, petrol, and bicycles, where cars depend on gasoline as fuel, and are obviously more closely related to each other than cars and bicycles, but it is generally believed that cars and bicycles are more similar to each other than cars and gasoline.

In an ontological structure, there is a similarity in the relationship between two concepts that are normally associated by an “is a”, and a correlation relationship between two concepts that are associated by other relationships (such as “part of”). It should be noted that semantic similarity or correlation is based on a perspective or context, and concepts that are similar or relevant in one perspective may not be similar or relevant in another.

However, in the conventional CBR decision-making system, the case database usually includes specific case knowledge, and not domain knowledge that is important for decision-making [14]. In addition, in traditional cases, the determination of similarity involves adopting a method based on keyword matching or keyword distance, with the semantic correlation relationship contained in the keyword itself not being considered. Therefore, the present study is devoted to improving case similarity matching and the CBR similarity degree algorithm, to acquire better matching cases.

In summary, the application of smart technology in the construction of safety risk management decision-making is a likely trend in future safety management. Based on the combination of ontology and CBR, it is easy to reuse and share knowledge in construction safety management. However, combining the two methods has received little attention in the existing construction safety risk management literature. The method used in this paper is highly improved for safety risk identification, reuse, and management by incorporating more relevant and useful information.

## 3. Research Methods

**Figure 1:**
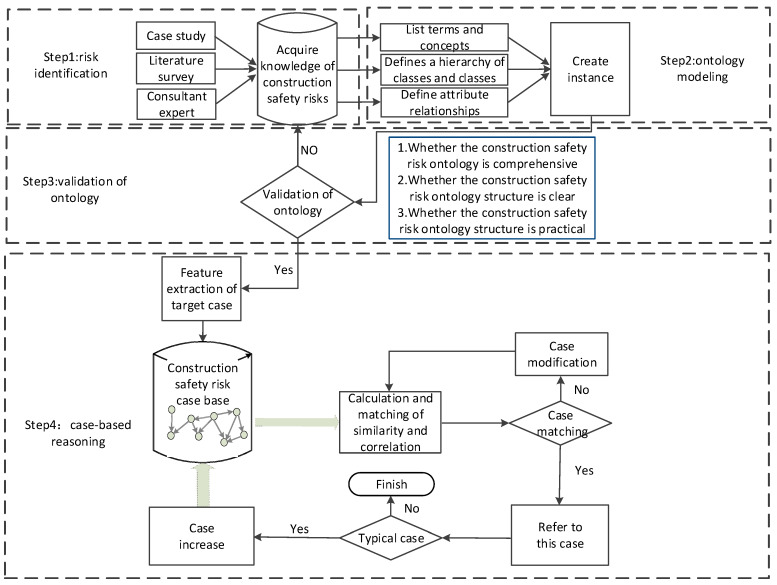
Construction safety risk management decision-making framework and implementation steps based on ontology and CBR.

This study develops a decision method for construction safety risk management based on ontology and CBR methods in which semantic similarity and a semantic correlation algorithms are combined. Figure 1 depicts the framework and implementation steps involved.

Step 1: Construction Safety Risk Identification

The construction safety risks were obtained from the literature, the case study, and the interviews from experts. Firstly, the literature related to construction safety risks was reviewed, and risks were summarized and categorized. Then, historical cases were investigated to extract the key construction safety risks involved. Finally, domain experts experienced in construction safety risk management evaluated the safety risks identified.

The reuse and sharing of the existing ontology in the construction field were also considered before constructing the new ontology. Generally, there are two methods for ontology reuse, one includes new ontology being retained as a part of an existing ontology and adds new knowledge, and the other is to extend the existing ontology by combining a new ontology with the same concepts and relationships of existing ontologies.

Step 2: Ontology Modeling

**Figure 2:**
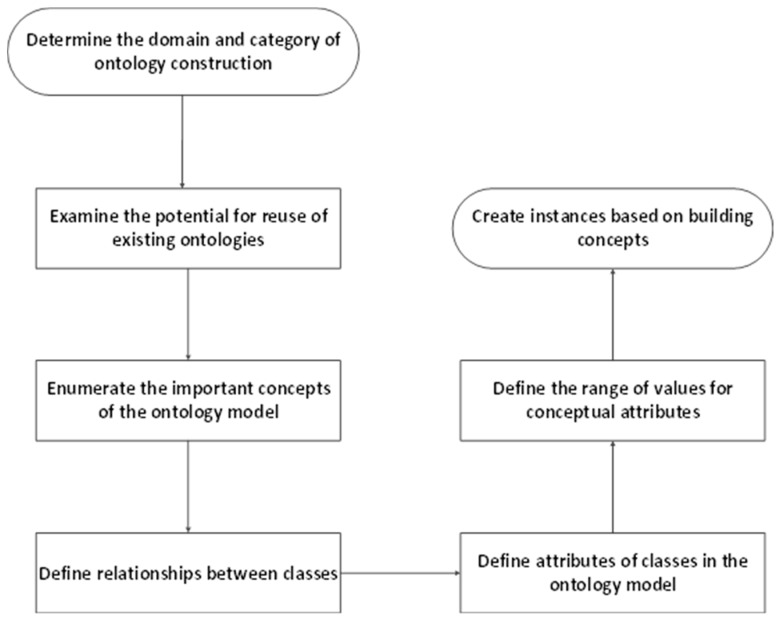
Seven-step construction process.

Protégé ontology modeling software is used to develop the construction safety risk ontology model, and an improved Seven-step method used as the ontology model development method. The steps are depicted in Figure 2.

Step 3: Ontology Verification

**Figure 3:**
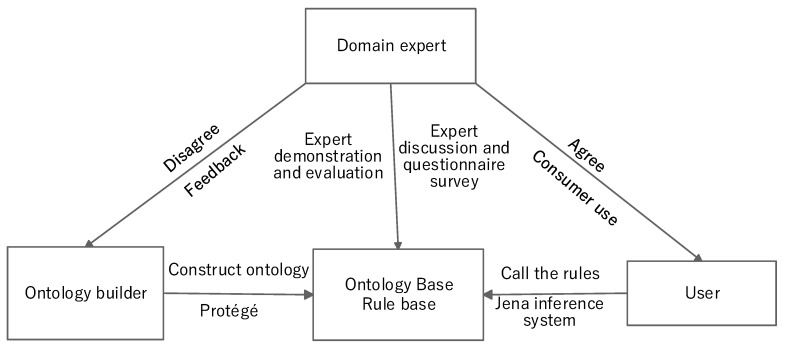
Validation of ontology.

It is necessary to evaluate and verify the developed construction safety risk ontology. Ontology verification requires the participation of domain experts of construction safety management to complete the optimization and modification of the ontology. The verification procedure is shown in Figure 3.

Step 4: Case-Based Reasoning (CBR)

Construction risk ontology provides a way for the standardized input of construction risk history cases, based on which, construction risk cases can be stored and reused effectively. The present study proposed an ontology-based comprehensive similarity and correlation method to conduct case reasoning. Before developing the safety risk case reasoning approach, past events or experiences were first stored in a case database using ontology technology. When a construction safety risk occurs, managers extract attributions of the case by referring to the ontology, and then compute the comprehensive similarity and correlation between the case base index and the target case index. The similarity of each index of the cases is weighted and summarized, and the similarity value between the cases is obtained. Then, one or several case’s risks with the highest similarity to the target case’s risks can be retrieved from the case base to provide a reference for the target case’s risk management. If the retrieved case is not applicable to the target case, modifications and adjustments are made according to the target situation to obtain a solution. Then, the target case is saved as a new case in the case database for subsequent retrieval.

CBR problem solving is therefore a four-phase process of retrieve, reuse, revise, and retain [75], the main purpose of which is to retrieve the most similar previous cases from the case base to solve a new problem. Therefore, this study mainly focuses on retrieval rather than the other three phases, and involves five steps: (1) calculating the attribute semantic similarities, (2) calculating the attribute semantic correlation, (3) calculating the comprehensive attribute semantic similarity and semantic correlation, (4) deriving the attribute weights of cases, and (5) calculating the weighted case similarities.

**Figure 4:**
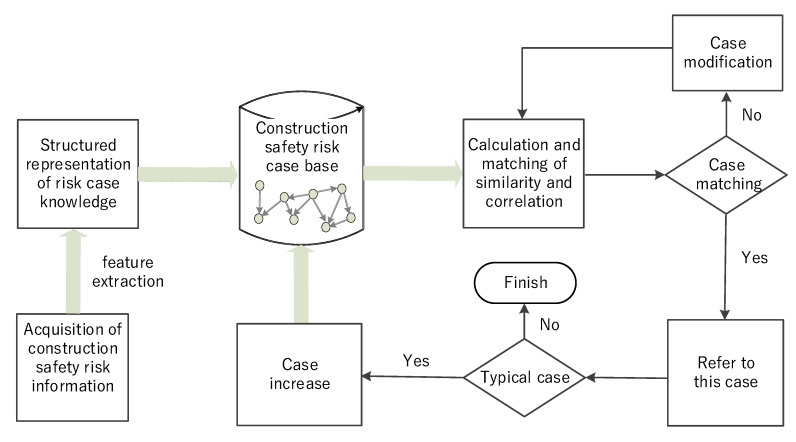
CBR framework for construction safety risks.

The risk case reasoning process is depicted in Figure 4.

### 3.1. Calculating the Attribute Semantic Similarities

#### 3.1.1. Semantic Similarity Algorithm Based on Distance

Generally, the semantic similarity algorithm based on distance determines the degree of similarity according to the distance between two concepts in a hierarchical network that is the ontology tree. These concepts are represented in the tree by nodes; the closer the distance, the higher the similarity. Assuming that a and b are two nodes in the tree, and defining the distance between the two nodes as dist(a,b), then

(1) $$dist\left(a,b\right)=\{\begin{array}{cc} 0 & a,b\;\mathrm{is}\;\mathrm{the}\;\mathrm{same}\;\mathrm{node}\;\mathrm{in}\;\mathrm{the}\;\mathrm{ontology}\;\mathrm{tree}\\ \infty & a,b\;\mathrm{have}\;\mathrm{no}\;\mathrm{common}\;\mathrm{ancestors}\;\mathrm{in}\;\mathrm{the}\;\mathrm{ontology}\;\mathrm{tree}\\ N & n\;\mathrm{is}\;\mathrm{the}\;\mathrm{sum}\;\mathrm{of}\;\mathrm{the}\;\mathrm{sides}\;\mathrm{of}\;a\;\mathrm{and}\;b \end{array}$$

the semantic similarity being obtained based on distance as

(2) $${\mathrm{Sim}}_{1}(a,b)=\{\begin{array}{ll} 1 & \mathrm{dist}\left(a,b\right)=0\\ 0 & \mathrm{dist}\left(a,b\right)=\infty \\ \frac{\partial \times N+1}{n+\partial \times N+1} & \mathrm{dist}\left(a,b\right)=n \end{array}$$

where ${\mathrm{Sim}}_{1}\left(a,b\right)$ indicates the similarity value of the concept represented by node a, the concept represented by node b, dist(a,b) is the distance between two nodes of a and b, and N is the distance between the root node of the construction safety risk ontology and the closest parent node of nodes a and b. ∂ is an adjustment factor that represents a domain expert’s opinion of the similarity degree and can be obtained by expert interview or questionnaire.

#### 3.1.2. Semantic Similarity Algorithm Based on the Hierarchy

In the ontology tree, the deeper the node is in the hierarchy of the ontology tree, the more specific concept it represents. Therefore, the depth of the node has an influence on the semantic similarity. A larger depth difference between the two nodes causes a smaller similarity.

Suppose that the hierarchy of node a in the ontology tree is D(a), the hierarchy of node b in the ontology tree is D(b), and the largest hierarchy in the ontology tree is D(C). Let $Sim_{2}\left(a,b\right)$ represent the similarity based on the hierarchy between nodes a and b. When a = b, it can be easily inferred that the attributed similarity is 1. Therefore, based on the research [76], the improved algorithm is

(3) $${\mathrm{Sim}}_{2}(a,b)=\left\{\begin{array}{ll} 1 & if\;a=b;\\ \frac{D\left(a\right)+D\left(b\right)}{\left|D\left(a\right)-D\left(b\right)\right|+2\times D\left(C\right)} & if\;a\neq b. \end{array}\right\}$$

#### 3.1.3. Semantic Similarity Algorithm Based on Content

Assuming that a and b are two nodes in the tree, C is the root node, P(a) represents the number of nodes between node a and C, and P(b) represents the number of nodes between node b and C. P(a) ⋂ P(b) and P(a) ⋃ P(b) respectively represent their intersection and union. Let $Sim_{3}\left(a,b\right)$ represent the semantic coincidence between a and b, with

(4) $${\mathrm{Sim}}_{3}(a,b)=\frac{\left|P(a)⋂P(b)\right|}{\left|P(a)⋃P(b)\right|}$$

#### 3.1.4. Comprehensive Attribute of the Semantic Similarity Algorithm

Considering the above three factors affecting semantic similarity, we propose the comprehensive semantic similarity algorithm

(5) $$Sim\left(a,b\right)=\omega_{1}\times {\mathrm{Sim}}_{1}(a,b)+\omega_{2}\times {\mathrm{Sim}}_{2}(a,b)+\omega_{3}\times {\mathrm{Sim}}_{3}\left(a,b\right)$$

where $\omega_{1},\;\omega_{2},\;\omega_{3}$ are weight parameters that can be obtained from expert experience, machine learning, or statistical methods, and $\omega_{1}+\omega_{2}+\omega_{3}=1$.

### 3.2. Calculating Semantic Correlation

In the ontology description language, there are two types of conceptual relationships: the attribute type (owl: Object Property) and data type (owl: Data Property). The data type represents the relationship between concepts and values, but it is not a problem of semantic correlation, which means that it is only necessary to consider the influence of the attribute type on the correlation of the ontology concept.

Definition: set any two nodes, a and b, in the ontology tree; S(a,b) indicates the shortest path length from a to b. Based on this, we can derive the correlation formula between the two concepts on the domain ontology as

(6) $$\mathrm{Cor}\left(a,b\right)=\left\{\begin{array}{ll} 1 & \mathrm{if}\;a=b\\ \frac{\omega_{4}}{S(a,b)+\omega_{4}} & \mathrm{if}\;a\neq b \end{array}\right\}$$

where $\omega_{4}$ is an adjustable parameter determined by the path length.

### 3.3. Calculating Comprehensive Semantic Similarity and Semantic Correlation

Semantic similarity represents the relationship between ontology and semantics, while semantic correlation represents the relationship between concepts. Combining these to examine the relationship between concepts gives

$$Sim^{*}\left(a,b\right)=\omega_{5}\times Sim\left(a,b\right)+\left(1-\omega_{5}\right)\times Cor\left(a,b\right)$$

where, $\omega_{5}$ is the weight parameter of semantic similarity in the similarity calculation that can be obtained by expert experience, machine learning, or statistical methods.

### 3.4. Deriving Index of Weights of Cases

In reality, there are a large number of indicators in the subway construction risk case; therefore, it is important to determine their weights. This is done here by a combination of expert evaluation and analytic hierarchy process (AHP) [77].

Firstly, this is done by establishing the indicator judgment matrix:

(7) $$X=\left[\begin{array}{cccc} x_{11} & x_{12} & \cdots & x_{1n}\\ x_{21} & x_{22} & \cdots & x_{2n}\\ \vdots & \vdots & \ddots & \vdots \\ x_{n1} & x_{n2} & \cdots & x_{\mathrm{nn}} \end{array}\right]$$

Each column in the matrix represents a certain kind of indicator, and $x_{ij}$ is the relative value of the indicator importance, which means the importance of the index in column i is relative to the index in column j. Secondly, the relative importance of the values of the indicators of each row are added to solve Equation (8), and then the sum of the relative values of the importance degrees of all the indices is obtained to solve Equation (9), with

(8) $$Y_{i}=\overset{n}{\underset{j=0}{\sum }}\;\;x_{ij}\left(j=1,2,\ldots ,n\right)$$

(9) $$U=\overset{n}{\underset{i=0}{\sum }}\;\;Y_{i}=Y_{1}+Y_{2}+\ldots Y_{n}$$

The weight of each attribute $p_{i}$ can be obtained by using the sum of the index importance relative values of each row $Y_{i}$ and then dividing the sum of index importance relative values of all indices U, as shown in Equation (10):

(10) $$p_{i}=\frac{Y_{i}}{U},p={\left(p_{1},p_{2}\ldots p_{n}\right)}^{T}$$

### 3.5. Calculating Case Similarity

Assume there are n cases in the subway risk case database, and each case has *m* attributed indicators. A similarity matrix is constructed so that each row in the matrix represents a case, and each column represents an attribute. The similarity matrix for each indicator of the target project and each indicator for the historical case in the case base is expressed as

(11) $$\mathrm{Sim}=\left[\begin{array}{cccc} \mathrm{Sim}\left(1,1\right) & \mathrm{Sim}\left(1,2\right) & \cdots & \mathrm{Sim}\left(1,m\right)\\ \mathrm{Sim}\left(2,1\right) & \mathrm{Sim}\left(2,2\right) & \cdots & \mathrm{Sim}\left(2,m\right)\\ \vdots & \vdots & \ddots & \vdots \\ \mathrm{Sim}\left(n,1\right) & \mathrm{Sim}\left(n,2\right) & \cdots & \mathrm{Sim}\left(n,m\right) \end{array}\right]$$

The index similarity is multiplied by the index weight to obtain the similarity q between the target and the historical case in the case base as

(12) $${{[q}_{1}\ldots q_{n}]}^{T}=\left[\begin{array}{cccc} \mathrm{Sim}\left(1,1\right) & \mathrm{Sim}\left(1,2\right) & \cdots & \mathrm{Sim}\left(1,m\right)\\ \mathrm{Sim}\left(2,1\right) & \mathrm{Sim}\left(2,2\right) & \cdots & \mathrm{Sim}\left(2,m\right)\\ \vdots & \vdots & \ddots & \vdots \\ \mathrm{Sim}\left(n,1\right) & \mathrm{Sim}\left(n,2\right) & \cdots & \mathrm{Sim}\left(n,m\right) \end{array}\right]\times \left[\begin{array}{c} p_{1}\\ p_{2}\\ \vdots \\ p_{m} \end{array}\right]$$

## 4. Case Verification

A subway construction project is used to demonstrate and verify the proposed construction safety risk management method based on ontology and CBR. The system is developed in Java language, using the Jena, JDK, MySQL, and protégé to build the ontology. Firstly, the model for the subway construction based on the safety risk ontology is completed in protégé and saved as an owl type file. Then, the risk ontology model file is stored and integrated into the MySQL database through Jena. After completing the integration of the ontology database, Jena is used to design a program for the similar case reasoning algorithm.

### 4.1. Construction Safety Risk Subway Project Identification

The subway project construction risks can be summarized based on a large number of studies and related historical cases, combined with the suggestions of subway area experts. The ontology mainly describes the potential risks in the subway construction process, which are mainly composed of risk types, sources, levels, consequences, and prevention measures [8].

In subway construction, there are two main types of risks. One is the technical risk caused by human operation (e.g., complex construction methods), and the other is natural environmental risk, such as the risk of a building collapse caused by poor geological conditions.

The source of risk is a description of the potential safety risks, mainly from environmental changes, equipment failures, or risks caused by human actions or operational errors. The subway construction risk source is defined here as a potential event that may induce risks in the project. At present, there is no clear classification of the risk sources for subway construction projects. An improved version of the method developed by Fidan et al. [78] is therefore used which proposes to divide engineering project risk sources into two categories of unexpected situations and adverse changes. The risk sources are first classified according to the type of risk, and the subcategories of unexpected and adverse changes are further supplemented. Table 3 provides an example of the environmental changes, equipment failures, human behavior, or operational errors involved.

The risk level is used to measure the severity of the risk. According to the probability of occurrence of risk events, risk loss, and social impact, the risk can be divided into five levels (Table 4).

The risk consequences are mainly used to indicate the consequences of the occurrence of risks, mainly involving casualties, economic losses, environmental damage, and construction shutdowns.

Risk prevention measures generally comprise three main types: design measures, construction measures, and management measures [8].

### 4.2. Subway Risk Ontology Modeling

**Figure 5:**
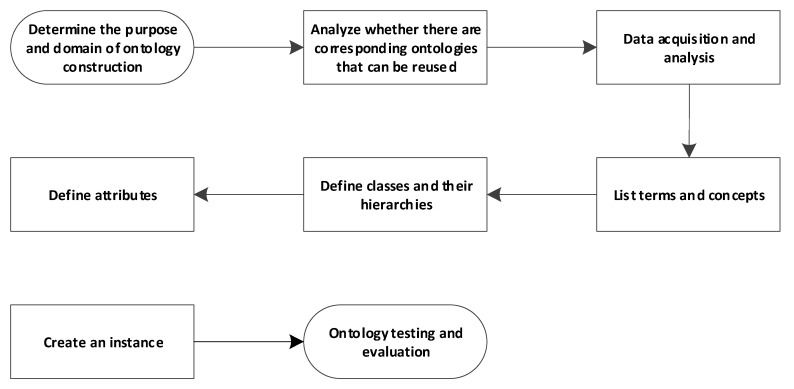
Development steps of the subway risk ontology.

Ontology modeling software protégé is used to construct the subway construction risk ontology based on the Seven-step method [43]; the ontology development steps are shown in Figure 5.

After determining the purpose and domain ontology, it is known whether there is an existing ontology that can be reused. The main difficulty in the modeling is to clarify the classes and attributes of the risks involved.

#### 4.2.1. Classes and Their Hierarchical Relationship

Five classes of risks are defined in the ontology, namely risk types, sources, levels, consequences, and prevention measures.

#### 4.2.2. Attribute Relationships

**Figure 6:**
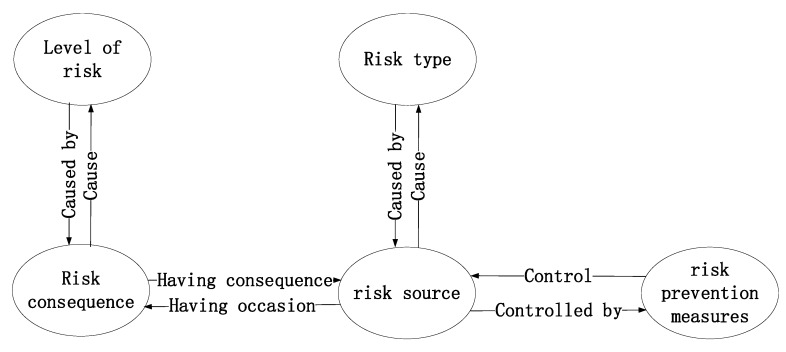
Attribute relationships.

The attribute can not only explain the situation of the ontology class in detail, but can also link the classes through defining the domain and range of the attribute, which is helpful for the intuitive input and representation of the safety cases. Such attributes as “Cause”, “Having consequence”, “Control”, and “Respond to”, are defined here. The attribute relationships between the various classes are shown in Figure 6.

**Figure 7:**
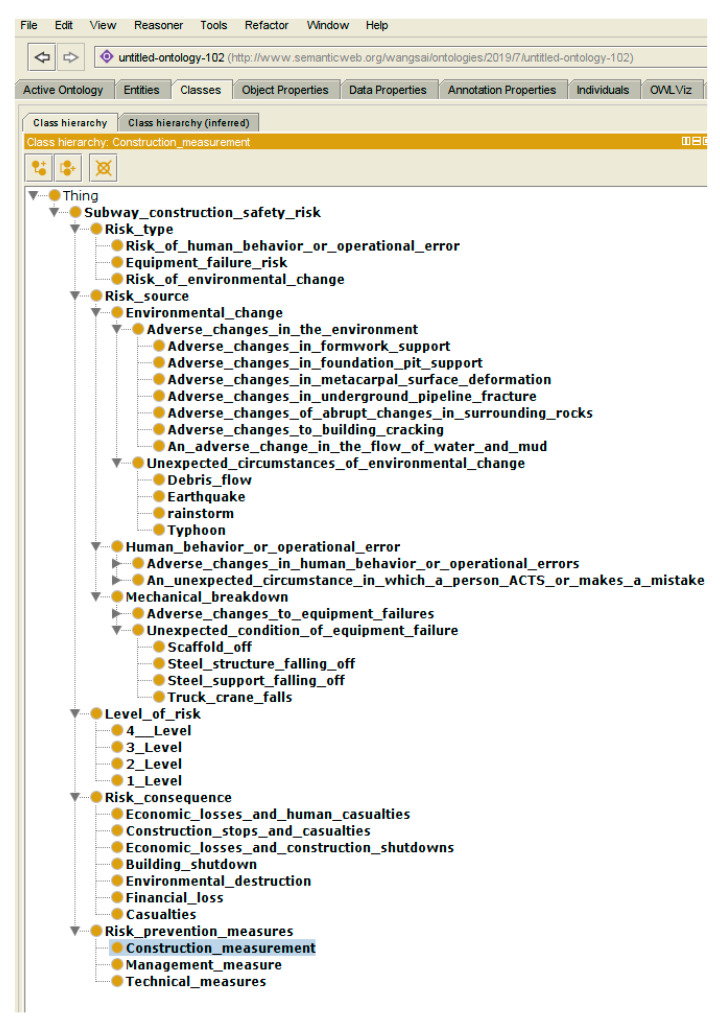

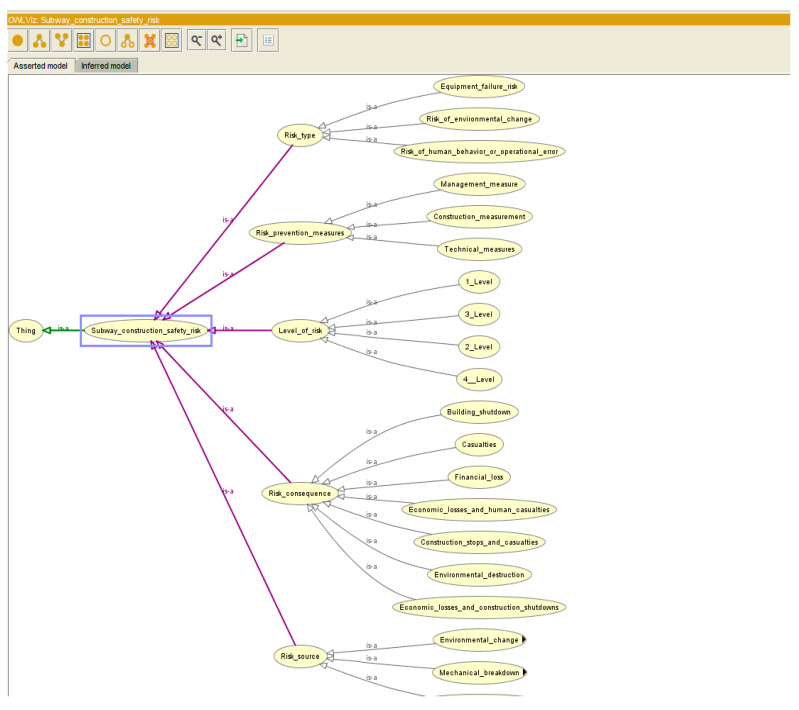
Subway construction safety ontology (section).

After the classes and attributes are determined, the ontology model is built as shown in Figure 7.

### 4.3. Ontology Verification

Ontology evaluation is essential for the development of ontologies [79]. Criteria-based evaluation is an important ontology evaluation method used to verify the content of ontologies through a set of predefined criteria [80]. Seminars are considered to be the main form of conducting criteria-based evaluation (the consistency criterion evaluation is based on a logic reasoner) [8]. Ten experts engaged in subway construction projects were interviewed for ontology verification. Firstly, the experts were informed of the relationship between classes and attributes, and then introduced to the whole decision system and reasoning process. Secondly, detailed discussions were held with the experts, and their recommendations were recorded in detail. Finally, the experts completed a questionnaire, and the ontology was revised accordingly. This involved a five-point Likert scale from 1 (strongly disagree) to 5 (strongly agree) to evaluate their level of agreement with three simple statements regarding the ontology. The results (Table 5) are taken to mean that the experts verify that the safety risk ontology and reasoning system of the subway project are comprehensive, concise, and practical.

### 4.4. Subway Case Reasoning

#### Construction of the Case Base

After the subway construction safety risk ontology is established, the base case is easy to compile. Five attributes are used comprising the risk source, type, level, consequence, and prevention measures as important indicators. The risk attribute indicators of the current project were extracted, the five most important being “environmental change”, “adverse water inrush changes”, “risk level is 4”, “economic loss and construction stoppage”, and “technical measures”. Assume the following five cases exist in the case base as shown in Table 6.

### 4.5. Reasoning Results

#### 4.5.1. Determination of Indicator Weights

The subway construction safety risk experts compared the risk types, sources, levels, consequences, and prevention measures mentioned above according to the importance of each indicator; the index judgment matrix being obtained from Equation (7) as

(13) $$X=\left[\begin{array}{ccccc} 1 & 6/5 & 7/9 & 11/10 & 1/2\\ 5/6 & 1 & 2 & 5/2 & 11/5\\ 9/7 & 1/2 & 1 & 10/11 & 5/11\\ 10/11 & 2/5 & 11/10 & 1 & 5/9\\ 2 & 5/11 & 11/5 & 9/5 & 1 \end{array}\right]$$

The sum of the importance of relative values of each row and the sum of index importance relative values of all indexes are calculated by Equations (8) and (9) as

(14) $$Y_{1}=4.58,Y_{2}=8.53,Y_{3}=4.14,Y_{4}=3.96,Y_{5}=7.45$$

(15) U=28.66

while the weights of each indicator are calculated by Equation (10) as

(16) p=[0.160 0.298 0.144 0.138 0.260 ]

#### 4.5.2. Case Similarity Correlation and Parameter Settings

Through the analysis of 83 Chinese subway construction statistics and correlations occurring in 2017–2018 (see Supplementary Material), the adjustment factor is set to $\omega_{1}=0.5,\omega_{2}=0.3,\omega_{3}=0.2,\;\mathrm{and}\;\omega_{5}=0.7$, calculated, as suggested in Gu et al [81], by Genetic Algorithm research. The attribute similarity indicators are listed in Table 7, Table 8 and Table 9 and the Semantic correlation calculation results is listed in Table 10.

The comprehensive similarity calculation is

(17) $$\begin{array}{c} \mathrm{sim}=0.7*\{0.5[\begin{array}{ccccc} \frac{3}{5} & \frac{3}{7} & 1 & \frac{3}{5} & 1\\ 1 & \frac{3}{5} & \frac{3}{5} & 1 & 1\\ 1 & \frac{3}{5} & \frac{3}{5} & \frac{3}{5} & \frac{3}{5}\\ \frac{3}{5} & \frac{3}{7} & 1 & \frac{3}{5} & \frac{3}{5}\\ \frac{3}{5} & \frac{3}{7} & \frac{3}{5} & 1 & \frac{3}{5} \end{array}]+0.3[\begin{array}{ccccc} \frac{3}{4} & \frac{3}{4} & 1 & \frac{3}{4} & 1\\ 1 & \frac{3}{4} & \frac{3}{4} & 1 & 1\\ 1 & \frac{3}{4} & \frac{3}{4} & \frac{3}{4} & \frac{3}{4}\\ \frac{3}{4} & \frac{3}{4} & 1 & \frac{3}{4} & \frac{3}{4}\\ \frac{3}{4} & \frac{3}{4} & \frac{3}{4} & 1 & \frac{3}{4} \end{array}]\\ \;+0.2[\begin{array}{ccccc} \frac{1}{2} & \frac{3}{5} & 1 & \frac{1}{2} & 1\\ 1 & \frac{3}{5} & \frac{1}{2} & 1 & 1\\ 1 & \frac{3}{5} & \frac{1}{2} & \frac{1}{2} & \frac{1}{2}\\ \frac{1}{2} & \frac{3}{5} & 1 & \frac{1}{2} & \frac{1}{2}\\ \frac{1}{2} & \frac{3}{5} & \frac{1}{2} & 1 & \frac{1}{2} \end{array}]\}+0.3*\{[\begin{array}{ccccc} \frac{3}{5} & \frac{3}{7} & 1 & \frac{3}{5} & 1\\ 1 & \frac{3}{5} & \frac{3}{5} & 1 & 1\\ 1 & \frac{3}{5} & \frac{3}{5} & \frac{3}{5} & \frac{3}{5}\\ \frac{3}{5} & \frac{3}{7} & 1 & \frac{3}{5} & \frac{3}{5}\\ \frac{3}{5} & \frac{3}{7} & \frac{3}{5} & 1 & \frac{3}{5} \end{array}]\} \end{array}$$

(18) $$=\left[\begin{array}{ccccc} 0.618 & 0.520 & 1 & 0.618 & 1\\ 1 & 0.632 & 0.618 & 1 & 1\\ 1 & 0.632 & 0.618 & 0.618 & 0.618\\ 0.618 & 0.520 & 1 & 0.618 & 0.618\\ 0.618 & 0.520 & 0.618 & 1 & 0.618 \end{array}\right]$$

(19) $${{[q}_{1}\ldots q_{n}]}^{T}=\left[\begin{array}{ccccc} 0.618 & 0.520 & 1 & 0.618 & 1\\ 1 & 0.632 & 0.618 & 1 & 1\\ 1 & 0.632 & 0.618 & 0.618 & 0.618\\ 0.618 & 0.520 & 1 & 0.618 & 0.618\\ 0.618 & 0.520 & 0.618 & 1 & 0.618 \end{array}\right]\cdot \left[\begin{array}{c} 0.160\\ 0.298\\ 0.144\\ 0.138\\ 0.260 \end{array}\right]=\left[\begin{array}{c} 0.743\\ 0.835\\ 0.683\\ 0.644\\ 0.647 \end{array}\right]$$

the current case two having the highest similarity (0.835) with the target project. Therefore, the risk treatment prevention of case two is taken as the main reference to the target case. Cases three, four, and five have low similarity, and will not be considered in decision-making.

## 5. Discussion

In previous studies, most semantic similarity algorithms have been considered based on attributes, content, and distance, of which the distance-based similarity algorithm is the most widely used [2,35,82]. However, solely using these algorithms can result in large errors and a better approach is to apply all three approaches simultaneously as is done here, that is, the hybrid similarity algorithm. The advantage of the algorithm is that it considers both the position information of the class in the ontology tree and the content information contained in the keyword itself.

The setting of attribute weight is a crucial step in this algorithm. Similarity-based attribute assessment involves the systematic comparison of the attributes of a target case and those of the previous cases stored in the case base. Different attribute weights can lead to different case similarities, and thus different previous cases may be retrieved for solving the target case. In such complex applications as subway safety risks, it is extremely difficult for human experts to quantify the relative importance of the attributes precisely. Such a weight elicitation process is subjective, and the weights elicited from different expert groups may differ greatly. In previous studies, various methods such as the equal weights method, feature counting, the gradient descent method, the analytical hierarchy process, decision trees, multiple regression analysis, neural networks, and genetic algorithms have been put forward to calculate attribute weight for CBR models to improve the estimation performance [83]. Weight learning of CBR is the future development trend within attribute selection and weight determination, such as evolutionary algorithms, entropy method, etc. [84,85,86]. Motivated by these previous studies, this paper adopts a genetic algorithm to calculate the attribute weight of the suggested CBR model.

Compared with semantic similarity, semantic correlation is neglected by many studies. Semantic similarity and correlation are two different concepts, semantic correlation referring to the degree to which two concepts are interrelated. A very common situation is that there may be no similar relationship between two concepts but they may be correlated for other reasons. Semantic similarity reflects the concept of aggregation, while semantic correlation represents the combination of concepts. This paper has made some explorations into the semantic correlation, but further improvements will be needed in the future.

## 6. Conclusions

In practice, construction engineering is a complicated process involving frequent risks. Construction safety risk management plays an important role in construction safety production. However, unstructured safety knowledge and cases are difficult to be identified, encoded, and reused, resulting in low efficiency risk management. In order to improve construction safety risk management and decision-making, this study proposes an ontology-based safety risk CBR method, which provides a more scientific method for managers to implement safety risk identification, reuse, and management.

Firstly, the integration of ontology technology and CBR is applied to the methodology of construction safety. Then, the similarity and correlation algorithms are integrated to improve the CBR algorithm, and the case with the highest similarity is found by calculating case similarity and correlation. We also verify the method through an application to subway construction safety risk management. Through a literature review and a case study, the study extracts the subway construction risk factors and establishes the ontology model of subway construction safety risk to put forward some measures of metro construction safety risk management to help construction safety risk management decision-making.

Semantic similarity and semantic correlation are considered to be simultaneous, with semantic similarity having three parts. The first refers to similarity based on attributes, mainly referring to the attribute information of two risk indicators. The second refers to content-based similarity, mainly referring to the possibility that the two risk indicators can be replaced without changing the risk consequences and risk management measures involved. The third is based on the similarity of distance; the smaller the semantic distance between two risk indicators, the closer their semantic similarity will be, and vice versa. Semantic correlation refers to the combinatorial relationship between two risk indicators. For example, in this study, there is no commonality between risk type and risk source, but risk source is determined by risk type.

The study is limited to the classes and attributes in the built-in subway construction risk information ontology model being unable to cover all the project risk information, the cases constructed in this system are limited and more comprehensive practical cases are needed, and only a framework and basic method for solving problems are provided—the development of a practical ontology model and inference system is a long and complicated process, and its implementation in practice require repeated modification and test evaluation. Therefore, further research is needed to improve the construction risk database; ontology model and case base requiring constant modification; demonstration and maintenance; the construction risk case reasoning, revision, and retention; the selection of target cases, with more complex target cases being used to verify the feasibility of the system based on ontology and case-based reasoning; and the similarity algorithm, with data mining, machine learning, and statistical methods combined with CBR to provide a more accurate similarity assessment.

## Figures and Tables

**Table 2:** Applications of Case-Based Reasoning (CBR) in construction safety management.

Method	Purpose	References
CBR and knowledge-based systems	Construction hazard identification	[16]
CBR with nearest-neighbor retrieval (NNR) search	Adjudicating construction industry occupational accidents	[15]
CBR	Automatic retrieval of subway operation safety risks	[60]
CBR and risk response strategies	Generate an actual response policy plan	[61]
CBR	Improve building maintenance management levels	[62]
A web-based CBR-RBR system	Active fall protection systems	[13]
CBR and the genetic algorithm	Improve safety performance	[63]
CBR and artificial neural networks	Estimate the severity of major engineering safety incidents	[64]

**Table 3:** Subway construction safety risk sources.

Risk Type	Risk Source Type	
Environmental change risk source	Accidents	Rainstorm
		Typhoon
		Debris flow
		Earthquake
	Adverse change	Unfavorable changes in foundation pit support
		Adverse changes in formwork support
		Adverse changes in building cracking
		Adverse changes in underground pipeline fracture
		Adverse changes in the deformation of the face
		Unfavorable changes in surrounding rock mutations
		Unfavorable changes in water inrush
Equipment failure risk source	Accidents	Scaffolding off
		Steel support shedding
		Steel structure falling
		Car hanging drop
	Adverse change	Unfavorable changes in tower crane overturning
		Adverse changes in the overturning condition of steel structures
		Unfavorable changes in the condition of car hoisting
		Improper use of mechanical equipment causes adverse changes in fire
Human behavior or operational error risk source	Accidents	Unfavorable changes in electrical equipment caused by electrical equipment failure
		Object blow
		Heat stroke accident
		Poisoning accident
	Adverse change	Unfavorable changes in construction cracks
		Unfavorable changes in the deformation of the face due to blasting errors
		Unfavorable changes in landslides caused by blasting errors
		Unfavorable changes in the test section are not set
		Adverse changes in the design of precipitation schemes
		Unfavorable changes in the disposal or disposal of confined water
		Unfavorable changes in improper placement of foundation pit support systems

**Table 4:** Subway construction safety risk sources.

Risk Level	Level 1	Level 2	Level 3	level 4	Level 5
Severity of risk	Negligible danger	General danger	Significant danger	High danger	Extreme danger

**Table 5:** Survey results.

Problem	Mean	Median	Variance	Results
Do you think that the risk ontology covers the main content of the subway construction project risk information?	2.36	3	0.41	Better comprehensiveness
Do you think the risk ontology structure is clear, concise, and easy to understand?	2.43	3	0.20	Better conciseness
In the future, are you willing to apply this technology to your work?	2.19	3	0.29	Good practicality

**Table 6:** Risk attribute indicators of the target case and historical cases in the case base.

Projects	Risk Type	Risk Source	Risk Level	Risk Consequences	Precautions
Current project	Environmental change	Unfavorable water inrush changes	4	Economic loss and construction stoppages	Technical measures
Case one	Equipment failure	Scaffolding off	4	Building downtime and casualties	Technical measures
Case two	Environmental change	Unfavorable changes in surrounding rock	5	Economic loss and construction stoppages	Technical measures
Case three	Environmental change	Collapse caused by blasting mistakes	3	Economic losses	Management measures
Case four	Human behavior or operational error	Deformation of the rock face caused by blasting mistakes	4	Economic loss and casualties	Management measures
Case five	Human behavior or operational error	Unfavorable changes in construction cracks	3	Economic loss and construction stoppages	Construction measures

**Table 7:** Distance-based semantic similarity calculation results.

Cases	Risk Type	Risk Source	Risk Level	Risk Consequences	Precautions
Case one	3/5	3/7	1	3/5	1
Case two	1	3/5	3/5	1	1
Case three	1	3/5	3/5	3/5	3/5
Case four	3/5	3/7	1	3/5	3/5
Case five	3/5	3/7	3/5	1	3/5

**Table 8:** Attribute-based semantic similarity calculation results.

Cases	Risk Type	Risk Source	Risk Level	Risk Consequences	Precautions
Case one	3/4	3/4	1	3/4	1
Case two	1	3/4	3/4	1	1
Case three	1	3/4	3/4	3/4	3/4
Case four	3/4	3/4	1	3/4	3/4
Case five	3/4	3/4	3/4	1	3/4

**Table 9:** Content-based semantic similarity calculation results.

Cases	Risk Type	Risk Source	Risk Level	Risk Consequences	Precautions
Case one	1/2	3/5	1	1/2	1
Case two	1	3/5	1/2	1	1
Case three	1	3/5	1/2	1/2	1/2
Case four	1/2	3/5	1	1/2	1/2
Case five	1/2	3/5	1/2	1	1/2

**Table 10:** Semantic correlation calculation results.

Cases	Risk Type	Risk Source	Risk Level	Risk Consequences	Precautions
Case one	1/3	1/5	1	1/3	1
Case two	1/2	1/3	1/3	1	1
Case three	1	1/3	1/3	1/3	1/3
Case four	1	1/5	1	1/3	1/3
Case five	1/2	1/5	1/3	1	1/3

## Supplementary Materials

The following are available online at https://www.mdpi.com/1660-4601/17/11/3928/s1, 83 cases on subway construction accidents in China from 2001 to 2019.
