# Supplementary material for: A Decision Method for Construction Safety Risk Management Based on Ontology and Improved CBR: Example of a Subway Project
Source: Int J Environ Res Public Health. 2020 Jun 1;17(11):3928. doi: 10.3390/ijerph17113928 (PMC7312838; doi:10.3390/ijerph17113928)
Supplement: Supplementary file 1 [file ijerph-17-03928-s001.pdf]

Appendix

83 cases on subway construction accidents in China from 2001 to 2019

NO.	Time	Project	Accident type	Cause	Consequence
（1）	25/05/2001	Earthwork site at Zhuzilin depot of Shenzhen subway	Collapse	The continuous scour of heavy rain caused the soil to loosen	1 dead and 1 injured
（2）	20/08/2001	Luban road station of Shanghai subway line 4	Landslide in the pit	Not enough precipitation	4 people buried and killed
（3）	19/04/2002	Shenzhen subway Guomao station	Mechanical injury	Traction wire rope broke into two pieces	2 dead and 4 injured
（4）	01/07/2003	Shanghai subway line 4	Quicksand	Improper command by the construction unit, on-site management personnel illegal construction, flaws in the construction plan, and supervision by the supervision unit	The three buildings were severely tilted, and the flood control wall partially collapsed, causing flooding of the cofferdam and the direct economic loss was estimated to be 150 million yuan
（5）	08/10/2003	Beijing subway line 5 Chongwenmen station	Overturn	Construction discipline was not strict, workers operate illegally	3 dead and 1 minor injury, direct economic loss of 297 thousand yuan
（6）	17/03/2004	Guangzhou subway line 3 Panyu Dashi station	Landslide in the pit	Muddy soil, heavy rainfall led to expansion and loosening of the ground around the shaft causing landslides	1 dead and delay of 5 days
（7）	01/04/2004	Guangzhou subway line 3 Liyu station	Underground continuous wall collapse	Continuous rainfall, water immersed in the concrete brick wall, caused slackening and caused landslides elsewhere	Subsidence occurred within a certain range of the surrounding area, and nearby residential houses were affected to some extent

（8）	2004.8.3	Guangzhou subway line 5	Gas pipe rupture	Gas leakage caused by mechanical equipment drilling through gas pipelines, and soon reached the explosion limit	Tens of thousands of residents were evacuated nearby
（9）	2004.9.25	Pazhou section, Xingang east road, Guangzhou subway line 2	Foundation pit collapse	Collapse caused by large amount of tap water pouring into foundation pit	Collapse area of over 400 square meters
（10）	2005.1.4	Dongdan station of Beijing subway line 5	Support instability		1 dead and 1 injured
（11）	2005.4.5	Bottom of Lishuiqiao river of Beijing subway line 5	near-miss	Upstream flood discharge	
（12）	2005.7.13	Window of the world station, Shenzhen subway line 2 test section	collapse	The geological structure was soil and sand, with unstable quicksand, and the construction disrupts the geological structure of the area	Shennan avenue eastbound main road forced to close due to subgrade break
（13）	2005.7.21	Guangzhou subway Haizhu Square	Foundation pit collapse	The construction and design were inconsistent, the geological conditions were complicated, and there was water seepage and mud seepage in the weak and strong weathered interlayer	Two adjacent buildings tilted to varying degrees, part of the wall cracked, and five workers were injured
（14）	2005.8.1	Beijing subway line 5 Heping west bridge	The crane collapsed	Problems with crane mechanical hydraulic equipment	1 passerby dead
（15）	2005.9.24	Interchange station for Beijing subway line 4 and 10	Road collapse	Leakage of pipelines creates cavities and water sacs; construction precipitation and ground disturbance disrupted soil stability	The gantry crane collapsed, caused a 5m long, 3m wide and 2m deep collapse pit
（16）	2005.9.27	Beijing Xizhimen subway station	Gas leak	The gas pipe was drilled through by workers	Serious gas leak accident

（17）	2005.11.3	Xinzao station of Guangzhou subway line 4	Mechanical fall	The cable set on one side of the tunnel wall dropped suddenly	2 dead and 1 injured seriously
（18）	2005.11.30	Beijing subway line 10 Olympic Branch line station	Collapse	Long-term leakage of sewage pipes forms water sacs, which seriously destabilizes the soil	One 14m water pipe was suspended, one 60cm water pipe was broken, and a dump truck was buried
（19）	2006.1.3	Hujialou station of Beijing subway line 10	Working face water gushing	Water pipe rupture, caused large-scale collapse	Collapse of part of the main paving road
（20）	2006.2.27	Beijing subway line 10	Mechanical fall	The rope of the crane equipment broke and the bucket dropped	3 dead
（21）	2006.4.24	Guangzhou subway line 5 QuZhuang station	Explosion	Duct explosion	1 dead and 3 injured seriously
（22）	2006.6.27	Beijing subway line 10	Collapse	After seepage, the coating will loosen and eventually cause collapse	1 dead
（23）	2006.8.2	Guangzhou subway line 3	Collapse	Weak soil around 1 cubic meter	1 dead and 2 injured
（24）	2006.11.20	Beijing subway line 4	Collapse	Sewage pipe leakage caused saturated soil and sand boil, caused the road to collapse	Subsidence pours into foundation pit
（25）	2007.2.5	Nanjing subway Line 2	Seepage collapse		The road caused a collapse of 60 square meters, and the collapse caused the natural gas pipeline to rupture and explode
（26）	2007.3.28	Beijing subway line 10 Suzhou street station	Collapse	When local landslides and cracks occur in the construction section, no safety protection measures have been formulated, caused a secondary collapse	6 dead

（27）	2007.5.28	Chating station of Nanjing subway line 2	Landslide in the pit	Loose soil caused by continuous rainfall	2 dead and 1 injured
（28）	2007.9.30	Shanghai subway line 9	Segment fall	Violation operation	1 dead
（29）	2007.12.16	Nanjing subway line 2	Road collapse	There was an unknown hole in the upper part of the tunnel, which was filled with hydrated silt. During the construction, water and silt poured into the tunnel and caused a collapse accident	Create a large pit 10m deep
（30）	2008.1.17	Guangzhou subway line 5	Gushing collapse	Geological situation was more complicated	100 square meters of ground collapsed, partial traffic closure of the Pearl River Bridge
（31）	2008.4.1	Shenzhen subway line 3	Template collapse	Violation operation	3 dead and 2 injured
（32）	2008.5	Guangzhou subway line 6	Gas explosion	On-site safety management flaws	3 dead and 6 injured seriously
（33）	2008.11.8	Nanjing subway line 1 south extension	Support instability	Stent collapse	7 injured
（34）	2008.11.15	Xianghu station of Hangzhou subway line 1	Foundation pit collapse	Construction unit illegal construction, risky operation, severe over-excavation of foundation pit	It caused a major accident with a length of 100m and a width of 50m in the construction area, causing 21 deaths, 1 serious injury, and 3 light injuries, with a direct economic loss of 49.62 million yuan
（35）	2009.1.8	Caoyang Road station of Shanghai subway line 11	Fire	On-site safety management flaws	1 dead and 3 injured
（36）	2010.7.14	Shanghai subway line 15	Support instability	Geological situation was more complicated, violation operation	2 dead and 8 injured, direct economic loss of 1.5494 million yuan

（37）	2010.8.1	Shenzhen subway Baoan center station No. 1	Foundation pit collapse	There were thick layers of miscellaneous fill and silt, and continuous rainfall results in saturation of the soil moisture outside the foundation pit	The surrounding area affected by the accident was about 630 square meters
（38）	2010.11.25	Xianghu station of Hangzhou subway line 1	Collapse	Geological situation was more complicated, violation operation	1 dead and 1 injured
（39）	2011.4.2	Beijing subway line 10	Object strike	The safety awareness of the workers was weak and on-site safety management flaws	1 dead
（40）	2011.5.6	Tianjin subway line 2	Road collapse	Shield captain's illegal operation, geological reasons, insufficient on-site rescue measures	Local segment deformation and cracking, two shield machines buried underground
（41）	2011.5.8	Shenzhen subway Daxin subway station	Biogas poisoning	The safety awareness of the workers was weak and on-site safety management flaws	2 dead, 1 seriously injured and 3 slightly injured
（42）	2011.8.18	Civil engineering of Shanghai subway line 11	Collapse	violation operation, on-site safety management flaws	2 dead and 4 seriously injured
（43）	2011.11.15	Beijing subway line 10 phase 2	Object strike	On-site safety management flaws	1 dead
（44）	2012.5.22	Qilizhuang station of Beijing subway line 9	Falling	The safety awareness of the workers was weak and on-site safety management flaws	1 dead and 1 seriously injured
（45）	2012.8.7	Wuhan subway line 2 phase 1	Object strike	violation operation, on-site safety management flaws	1 dead
（46）	2012.8.23	Dongcheng district, Beijing subway line 7	Mechanical injury	On-site safety management flaws	1 dead
（47）	2012.11.9	Nanchang subway line 1	Collapse	violation operation	1 dead
（48）	2013.5.6	Xian subway line 3	Collapse	Geological situation was more complicated, violation operation	5 dead

（49）	2013.5.6	Beijing subway line 7	Mechanical injury	On-site safety management flaws	1 dead
（50）	2013.6.13	Changchun subway line 1	Collapse	Geological situation was more complicated, violation operation	1 dead
（51）	2013.9.4	Wuhan subway line 4	Mechanical injury	violation operation, on-site safety management flaws	1 dead and 2 seriously injured
（52）	2013.9.18	Wuhan subway line 4 phase 1	Electric	The safety awareness of the workers was weak and on-site safety management flaws	2 dead
（53）	2014.1.18	Yan'an third road station, Qingdao subway project	Mechanical injury	violation operation	1 dead
（54）	2014.3.7	Qingdao subway line 2	Object strike	The safety awareness of the workers was weak and on-site safety management flaws	1 dead
（55）	2014.3.23	Beijing subway Changping line phase 2	Collapse	Geological situation was more complicated	1 dead
（56）	2014.8.10	Zhengzhou subway line 2	Object strike	The safety awareness of the workers was weak	1 dead
（57）	2014.8.11	Guiyang subway line 1	Collapse	Geological situation was more complicated, violation operation	1 dead
（58）	2014.10.7	Nanning subway line 1	Foundation pit collapse	Natural weather influence	3 dead, direct economic loss of about 13 million yuan
（59）	2014.10.9	Qingdao subway line 2 phase 1	Mechanical injury	violation operation, defects in construction technology management	1 dead
（60）	2014.12.17	Nanjing subway line 4	Collapse of the steel frame	violation operation, defects in construction technology management	4 dead and 3 injured
（61）	2014.12.24	Wuhan subway line 3	Shield machine flooded	Burst of water supply pipe	It took three and a half months from submergence to recovery, with an

					economic loss of about 30 million yuan
（62）	2015.2.6	Qingdao subway line 2	Collapse	Geological situation was more complicated, violation operation	
（63）	2015.3.28	Nanjing subway line 4	Ground subsidence	Geological situation was more complicated	Three residential buildings subsidence
（64）	2015.5.11	Shenyang subway line 10	Collapse	Natural weather influence, geological situation was more complicated	1 dead
（65）	2015.6.25	Shenzhen subway line 7	Collapse	Geological situation was more complicated	1 dead and 4 injured
（66）	2015.8.28	Hangzhou subway line 4	Gas pipe rupture	The distribution map of underground pipelines at the construction section was inconsistent with the actual situation	Leakage 4300 cubic meters
（67）	2016.1.5	Wuxi subway line 3	Collapse	It failed to meet the requirements of 1: 1 slope set of construction organization design, and no other supporting measures were taken, resulting in insufficient stability of the slope	1 dead
（68）	2016.3.5	Shenzhen subway line 9	Collapse	The fill layer was mainly composed of cohesive soil, sand, gravel and a small amount of construction waste	1 dead and 1 injured, direct economic loss of 1.9 million yuan
（69）	2016.6.26	Shenzhen subway line 9	Falling	The safety awareness of the workers was weak and on-site safety management flaws	1 dead, direct economic loss of 1.6 million yuan
（70）	2016.7.8	Hangzhou subway line 4	Foundation pit soil surge	Geological situation was more complicated	4 dead
（71）	2016.7.29	Chongqing subway line 5	Collapse	violation operation, defects in construction technology management	3 dead and 1 injured, direct economic

					loss of 6.68 million yuan
（72）	2016.10.19	Shenyang subway line 9	Collapse	Geological situation was more complicated, violation operation	3 dead
（73）	2017.4.19	Shenzhen subway line 8	Collapse of the steel frame	violation operation, defects in construction technology management	1 dead, 1 seriously injured and 2 slightly injured
（74）	2017.5.11	Shenzhen subway line 3 phase 3	Collapse	Earthwork excavation was not carried out in accordance with the construction scheme	3 dead and 1 injured, direct economic loss of 3.45 million yuan
（75）	2018.1.26	Guangzhou subway line 21	Tunnel collapse	Geological situation was more complicated, violation operation	3 dead
（76）	2018.1.29	Chengdu subway line 5	Poisoning and suffocation	violation operation	2 dead and 1 missed
（77）	2018.2.7	Foshan subway line 2	Foundation pit collapse	There was a deep water-rich silt layer near the medium-coarse sand layer with strong water permeability	11 dead, 1 missed and 8 injured, direct economic loss was about 53.238 million yuan
（78）	2018.8.8	Guiyang subway line 2 phase 1	Collapse of secondary lining steel bars	Geological situation was more complicated	3 dead
（79）	2019.5.9	Shenzhen subway line 8 phase 1	Mechanical injury	violation operation	1 dead
（80）	2019.5.13	Shenzhen subway line 8 phase 1	Object strike	The safety awareness of the workers was weak and on-site safety management flaws	1 dead
（81）	2019.5.27	Qingdao subway line 4	Collapse	There were a lot of loose and weak strata such as backfill soft soil, sand soil and silt in the filling layer	5 dead

（82）	2019.5.28	Changzhou subway line 2 phase 1	Collapse	Geological situation was more complicated	1 dead
（83）	2019.06.23	Tianjin subway line B1 phase 1	Mechanical injury	violation operation	1 dead
